# Identification and Functional Characterization of Gene Components of Type VI Secretion System in Bacterial Genomes

**DOI:** 10.1371/journal.pone.0002955

**Published:** 2008-08-13

**Authors:** Sakshi Shrivastava, Sharmila S. Mande

**Affiliations:** Bio-Sciences Division, Advanced Technology Centre, Tata Consultancy Services, Madhapur, Andhra Pradesh, India; University of Pennsylvania School of Medicine, United States of America

## Abstract

A new secretion system, called the Type VI Secretion system (T6SS), was recently reported in *Vibrio cholerae, Pseudomonas aeruginosa and Burkholderia mallei*. A total of 18 genes have been identified to be belonging to this secretion system in *V. cholerae*. Here we attempt to identify presence of T6SS in other bacterial genomes. This includes identification of orthologous sequences, conserved motifs, domains, families, 3D folds, genomic islands containing T6SS components, phylogenetic profiles and protein-protein association of these components. Our analysis indicates presence of T6SS in 42 bacteria and its absence in most of their non-pathogenic species, suggesting the role of T6SS in imparting pathogenicity to an organism. Analysis of genomic regions containing T6SS components, phylogenetic profiles and protein-protein association of T6SS components indicate few additional genes which could be involved in this secretion system. Based on our studies, functional annotations were assigned to most of the components. Except one of the genes, we could group all the other genes of T6SS into those belonging to the puncturing device, and those located in the outer membrane, transmembrane and inner membrane. Based on our analysis, we have proposed a model of T6SS and have compared the same with the other bacterial secretion systems.

## Introduction

Pathogenicity of bacteria is often critically dependent upon machineries which mediate the transport of toxic molecules out of their cells into the environment or into the host cells [1]. These machineries are specialized systems, called the Secretion Systems. Six such secretion systems have been identified thus far, Type I through Type VI [1], which carry out the process of translocation, type VI being the most recent discovery [2], [3], [4]. All the secretion systems possess specialized components that mediate transport of effector proteins across the various membranes of bacteria, namely the inner membrane, the periplasmic space and the outer membrane. While type II secretion system (T2SS), and type V secretion system (T5SS) use the Sec translocase and N-terminal signal sequences for the transport of their effector proteins, type I (T1SS), type III (T3SS), type IV (T4SS) and type VI (T6SS) use sec-independent mechanism.

Since many of the secreted proteins are virulence factors, a complete understanding of the mechanism underlying bacterial protein secretion is crucial for understanding the host-pathogen association. The mechanistic details of T6SS are less well understood due to its recent discovery. T6SS has been reported in *Vibrio cholerae, Pseudomonas aeruginosa* and *Burkholderia mallei* [2], [3], [4]. Out of the 18 genes in T6SS in *V. cholerae,* 4 are thought to be a part of the structural components, 2 are effector molecules and 1 is a chaperone [2]. In *P. aeruginosa,* a triggering mechanism of the T6SS has been proposed where a serine-threonine phosphorylation event has been considered to be essential in bringing about the activation of the T6SS system [3]. Similarly in *B. mallei*, it has been observed that there are three T6SS clusters and the regulation of these is carried out by v*ir*AG, a two component regulatory system [4].

Although the genes which play a central role in carrying out secretion by T6SS pathway are known in *V. cholerae, P. aeruginosa* and *B. mallei* and show a conserved pattern in terms of sequence similarity, the mechanistic details of the machinery are not very clear. In order to understand the functioning of T6SS as well as to predict this system in other bacterial species, we have carried out an analysis using informatics approaches and results are presented in this paper.

## Results

### Orthology Search and Phylogenetic Profile Analysis

Table 1 lists all the organisms, having orthologs of at least 10 T6SS components of one or more of the three organisms, namely *V. cholerae, P. aeruginosa* and *B. mallei.* The bit scores and E-values of all the orthologs obtained are listed in the Table S1. In *V. cholerae,* genes comprising of the Virulence Associated (VAS) cluster, namely, *vasH*, *vasK*, *vasF* and *vasA* have been suggested to be of structural importance and critical for the T6SS machinery [1]. Effector proteins VgrG and Hcp, also present on the VAS cluster, are thought to be important not only as secreted products but also as part of the structural machinery [2]. The functions of the remaining genes are not clearly known. Results of the orthology study indicated that these four structural VAS genes and the two effector proteins are conserved in all 42 organisms. The other conserved proteins included a chaperone ClpB whose exact functional role in T6SS is not known, VCA0111, VCA0112, VCA0113, VCA0114, VCA0107 and VCA0108. Their high conservation suggested that these proteins could be the major requirement for a functional T6SS. On the other hand, the proteins VCA0118, VCA0121 and VCA0122 were seen to be restricted to a smaller group of organisms (Table S2) and could have a species specific role in the T6SS machinery of these organisms.

**Table 1:** List of organisms having orthologs of at least 10 components of known Type VI Secretion System. Organisms in bold are known to have T6SS.

*Organisms*	Number of T6SS orthologs
***Vibrio cholerae***	18
*Vibrio fulvinicus*	18
*Vibrio parahemolyticus*	18
*Erwinia caratovora*	17
*Yersinia pestis*	17
*Yersinia pseudotuberculosis*	17
*Yersinia enterocolitica*	17
*Escherishia coli O157*	17
*Escherishia coli B171*	18
*Escherishia coli CFT073*	15
*Escherishia coli 536*	18
*Escherishia coli APEC*	18
*Escherishia coli UTI189*	18
*Aeromonas hydrophila*	16
*Aeromonas salmonicida*	16
*Shigella sonnei*	16
*Shigella flexneri* 1	13
*Shigella dysentriae* 1	13
***Pseudomonas aeruginosa***	16
*Pseudomonas syringae*	16
*Pseudomonas putida*	16
*Pseudomonas fluorescens*	16
*Pseudomonas putida*	16
*Photorhabdus luminescens*	16
*Marinobacter aquaeolei*	15
*Mesorhizobium lot*	15
*Photobacteria profundum*	15
*Xanthomonas campestris*	14
*Xanthomonas oryzae*	14
*Xanthomonas axonpodis*	14
*Ralstonia metallidurans*	14
*Ralstonia eutropa*	14
*Ralstonia solanacearum*	14
*Hahella chejuenensis*	14
***Burkholderia mallei***	14
*Burkholderia pseudomallei*	14
*Burkholderia cepia*	14
*Burkholderia cenocepia*	14
*Geobacter mettaliduricans*	12
*Geobacter sulfurreducens*	12
*Salmonella enterica*	12
*Shewanella frigidamarina*	10

1 species picked up during the 2nd Blast search

The analysis of Blast search using the sequences of *V. cholerae, P. aeruginosa* and *B. mallei* showed that in case of Shigella, *Shigella sonnei* showed the presence of T6SS orthologs. However, orthologs in *Shigella dysentriae* and *Shigella flexneri* were detected only when a blast of T6SS orthologs of *S. sonnei* was done against all other Shigella species.

Analysis of completely sequenced genomes of various species and strains of each of the organisms appeared to suggest that the avirulent species lacked T6SS orthologs in most of the organisms (Table S3). For example, in *Escherichia coli*, the strain K12 did not show orthologs of any of the T6SS proteins, whereas all the other virulent strains, namely, O157, B171, 536, UTI89, APEC and CFT073, showed orthologs of all the T6SS components, with identities ranging from 90 to 99%. Similarly, in Vibrio, *Vibrio fischeri and Vibrio harvey,* which are avirluent, did not show any orthologs of the T6SS components whereas the virulent *V. cholerae, Vibrio parahemolyticus and Vibrio fulvinicus* showed orthologs of all the 18 components of T6SS. In Burkholderia too, the avirluent *Burkholderia thailandensis* did not show any T6SS orthologs whereas the virulent species had orthologs of 14 of the T6SS components. In Shigella it was seen that the virulent species *S. sonnei, S. flexneri and S. dysentriae* contained 18, 13 and 13 T6SS orthologs respectively, whereas the avirulent species *Shigella boydii* did not show any orthologs. The plant pathogen Xanthomonas also followed similar trend, where avirulent *Xanthomonas populi* and *Xanthomonas codiaii* lacked any orthologs. These results suggested strongly that T6SS could play a crucial role in imparting pathogenicity to an organism.

Species specific study (Table S3) also indicated that organisms which lacked one or more of the T6SS orthologs mostly lacked orthologs of VCA0118, VCA0119, VCA0121 and VCA0122. For example, orthologs of these proteins were absent in *S. flexneri and S. dysentriae,* each of which had 13 out of 18 orthologs. Similarly, *E. coli* CFT073 with 15 orthologs and all the species of Burkholderia and Xanthomonas, with 14 components each, also lacked these 4 genes.

**Figure 1:**
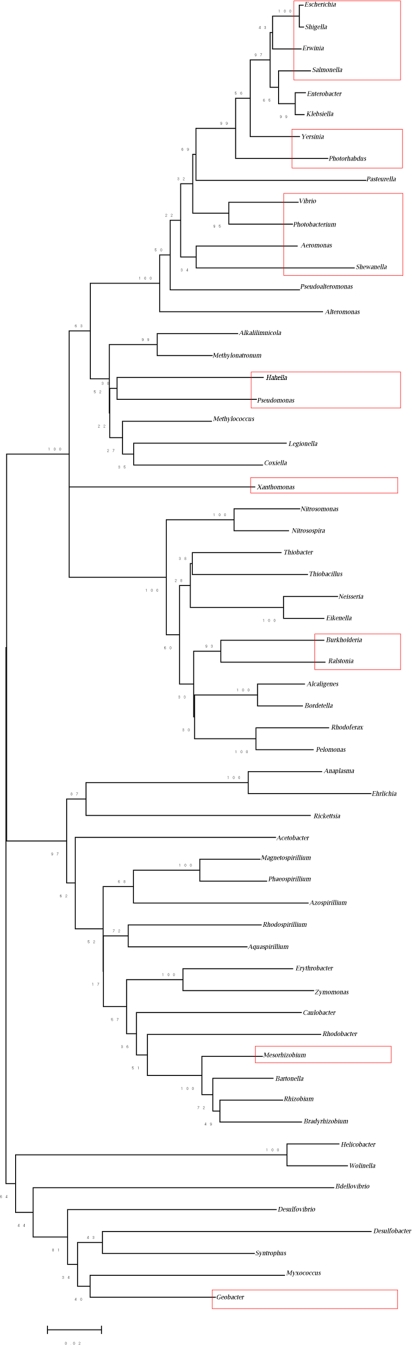
16S rRNA tree of proteobacteria with representative members from each family whose complete genome sequence is known. Boxed regions indicate organisms predicted to have Type VI Secretion System components. The tree was bootstrapped 500 times using *MEGA* 4.0 and the bootstrap values are shown in the tree.

All the bacterial species identified to have T6SS components belonged to the proteobacteria group of the gram negative pathogens (Figure 1). Among the gamma proteobacteria, few members of vibrionaceae, enterobacteriaceae, xanthomonaceae and pseudomonaceae families showed T6SS orthologs. Similarly, Ralstonia and Burkholderia, belonging to the beta proteobacteria, also exhibited T6SS genes. On the other hand, Geobacter and *Mesorhizobium loti,* belonging to the delta and the alpha Proteobacteria respectively, possessed T6SS. Thus, representatives of all the proteobacteria sub-groups (alpha, beta, delta and gamma) showed T6SS components; gamma proteobacteriaceae being the most widely represented one.

**Figure 2:**
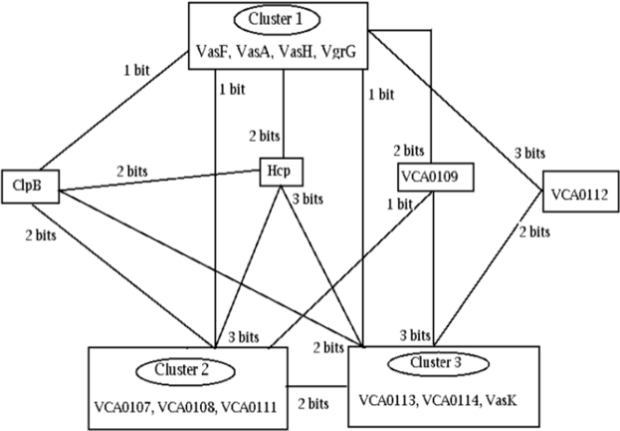
Schematic representation of gene clusters of Type VI Secretion System components having similar phylogenetic profiles.

On analyzing the phylogenetic profiles of T6SS proteins (Table S2), three significant protein clusters (proteins with similar profiles) were obtained. Each of these clusters differed from each other by a few bits ranging from one to three (Figure 2), suggesting relatedness in their functions. T6SS components belonging to the 3 clusters are given below.

**Cluster 1:** VasA, VasF, VasH and VgrG–All these proteins were present across all the organisms.

**Cluster 2:** VCA0107, VCA0108 and VCA0111-These proteins were present across all organisms except in Shewanella.

**Cluster 3:** VCA0113, VasK, VCA0114–These were absent only in Geobacter.

Cluster 1 differed from clusters 2 and 3 by one bit whereas clusters 2 and 3 differed from each other by two bits. Some of the proteins, namely ClpB, Hcp, VCA0109, and VCA0112 did not cluster with any other proteins. However they were seen to be located in the neighborhood space (defined in the methods section) of the above three clusters (Table S2). For example, Hcp differed from cluster 1 by two bits and clusters 2 and 3 by three bits each (Figure 2, Table S2). Similarly, VCA0109 was two bits different from cluster 1, one bit different from cluster 2 and three bits different from cluster 3. VCA0112 was three bit different to cluster 1 and two bits different to cluster 3. ClpB was one bit different from cluster 1 and two bits different from clusters 2 and 3. Thus all these proteins were located in the neighborhood space of each other implying functional linkage [5], [6]. Profiles of the proteins VCA0118, VCA0119, VCA0121 and VCA0122 were very different from the rest, and did not belong to any of the clusters, indicating that they were not represented uniformly across the organisms.

An analysis performed on all other proteins belonging to the T6SS neighborhood space revealed few additional genes in these organisms. This included OmpA, a protein present in all the organisms and whose phylogenetic profile matched with that of cluster 1. This suggested that OmpA, which is an outer membrane protein and is known to be involved in the formation of a translocation pore [7], might have functional relationship with the proteins belonging to cluster 1. Similarly, ClpV was identified to have similar profile as ClpB in all organisms, suggesting a functional relationship between these two proteins. Based on Pfam annotation ClpV is known to be an adapter protein to ClpB, reinforcing our observation about the functional relationship between these two proteins.

### Protein Domain Search

Information retrieved from the domain annotation indicated a number of genes possessing the DUF (Domains with Unannotated Functions) and the UCP (UnCharacterized Protein) domains. However some of the others had domains like the transmembrane domain, porin outer membrane domain, ImpA related domain, etc. For instance, ClpB exhibited an ATP domain and a chaperonin domain, indicating its role as an ATPase as well as a chaperone for the T6SS proteins. Similarly, VasH belonged to sigma-54 domain containing protein which is implicated in the transcriptional regulation of the T6SS gene cluster. VgrG had three major domains, namely, a peptidoglycan binding domain, a gp-5 domain and an Actin Cross linking Domain (ACD). Another protein called Hcp, contained a domain corresponding to the hemolysin-coregulated protein. Likewise, while VasF had a transmembrane domain, VasK was seen to be a IcmF like protein having a transmembrane domain. The hypothetical proteins, VCA0109 and VCA0112 contained a gp-25 domain acting as a lysozyme and a SMAD_FHA domain containing protein respectively. Similarly hypothetical proteins VCA0119 and VCA0121 had ImpA domains. All the information regarding the domains for each of the other T6SS components is given in Table S4.

### Family and Superfamily Search

Family and superfamily searches also revealed information about some of the proteins which included ClpB, VasH, VgrG, VasK, VCA0109, VCA0112 and VCA0121. ClpB belonged to a ATPase family associated with various cellular activities (AAA). Hcp belonged to a hemolysin coregulated-protein family of virulence factors for secretion apparatus. VasH belonged to the P-loop containing superfamily having a ATP/GTP binding motif and was also indicated to be a part of a homeodomain-like superfamily, which are implied in activation of transcriptional regulation. Similarly, VgrG, was seen to be a part of phage fibre protein superfamily, containing a gp-5 lysozyme at its C-terminal end and a peptidoglycan binding domain. VasK was also seen to be a part of the P-loop containing superfamily and a G-proteins family, suggesting its role in signal transduction. The hypothetical proteins VCA0109 and VCA0112 belonged to a Gene25-like lysozyme family and a SMAD/FHA domain containing superfamily respectively. The SMAD/FHA domain containing superfamily is known to carry a nuclear signaling domain which interacts with serine-threonine kinase receptors. Similarly the hypothetical protein, VCA0121 belonged to an ImpA related N terminal family which is known to have inner membrane proteins participating in intracellular protein transport.

### Motif Search

The search for motifs could not detect any known motifs in most of the T6SS proteins. Only ClpB and VasH had the motif specific for ATPase and Sigma54 respectively, suggesting their roles as a ATPase and a transcriptional regulator respectively.

### Signal Peptide Search

All the orthologs of the effector molecules of the T6SS lacked the cannonical N-terminal signal peptide, as predicted by SignalP [8]. This is in accordance with the suggestion by Pukatzki *et al.* [2] that T6SS is known to transport only those proteins which lack an N-terminal signal peptide. However, all the VgrG protein orthologs showed a C-terminal signal. It is seen that VgrG protein uses its C-terminal end to bring about cross linking via its actin crosslinking domain (ACD) and is responsible for the cross-linking activities with the host cell [9]. This is in contrast to that of the effector molecules of the T2SS and T5SS, where the N-terminal signal peptides in these molecules gets cleaved during the secretion process [9].

### Detection of Protein Folds of T6SS Components

Proteins which could be annotated based on their 3D folds included ClpB, Hcp, VgrG, VasF, VCA0109, VCA0112, VCA0119 and VCA0121. The three dimensional fold of ClpB matched with the folds of ClpA ATP-dependent Clp protease and to chaperone Hsp104 and related ATP-dependent Clp proteases. VasH showed remote homology to the fold of various transcriptional regulatory proteins, including that of two component system. VgrG protein showed homology to a vgr_GE Rhs element Vgr protein and also to the N-terminal domain of the tail-associated lysozyme, gp5. Similarly, the folds of hypothetical proteins, VCA0121 and VCA0119 matched with the VHS domain known to have a role in general membrane targeting and recognition as well as in protein trafficking. In addition, VCA0119 had a TPR like repeat domain which is known to be involved in protein-protein interactions and is known to mediate assembly of multiprotein complexes. While, the hypothetical protein VCA0112 showed similarity to a phosphoprotein/serine-threonine kinase binding domain, VCA0109 had a remote homology to the phage base plate assembly protein. The three dimensional fold of VCA0108 and VCA0107 showed some similarity to phage tail sheath protein and a protein ImpB implicated in protein secretion respectively. VCA0111 showed remote homology to a phage tail protein domain. Coiled coil structures were predicted for VasK and the hypothetical protein VCA0118. These two proteins were also predicted to be transmembrane proteins from our domain and family searches.

### Protein-Protein Association

Analysis of the associating partners of T6SS components revealed that a search against any one of the T6SS genes (in each organism) picked up all the 18 genes of the putative T6SS cluster of that organism, as its functional interacting partners. The prediction suggested that these proteins participate in a common pathway and function together. There were two additional proteins, VCA0893 and OmpA, which were not a part of the T6SS cluster but were seen to be associated with the T6SS genes. While VCA0893 is a chemotaxis protein, OmpA is a structural protein of the outer membrane which connects the outer membrane to the periplasmic peptidoglycan via its periplasmic domain.

### Location of T6SS Components in the Genome

Secretion systems have often been found to be located in the Pathogenicity Islands of the organisms [10], [11]. The predicted T6SS gene clusters were found to be located in the known pathogenicity islands in two of the organisms, namely, *E.coli BEN2908 AGI-1* AY395687 and *Photorhabdus luminiscens-W-14 Mt Locus AY144117.*

**Figure 3:**
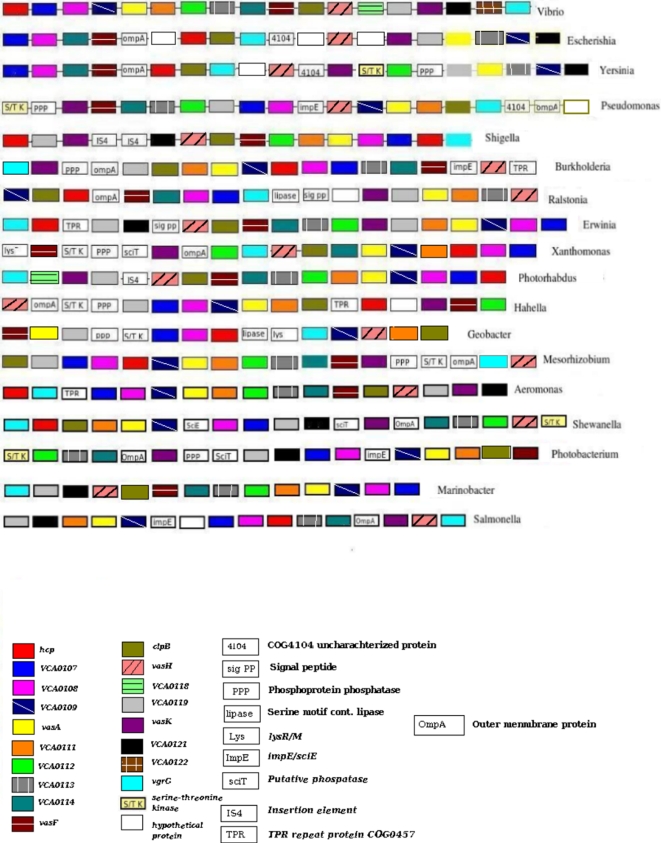
Genomic Islands containing Type VI Secretion System components. The order of the T6SS genes as they occur in the respective genomes is shown. Only one species of each organism is represented. The gene (T6SS components) names correspond to those in *V. cholerae*. Additional genes which are not known to be part of the T6SS gene clusters are also highlighted.

The predicted T6SS gene cluster in most of the organisms was found to be located in compositional distinct regions of the genomes (Genomic Islands) as identified by the centroid method [11] **.** Apart from the identified T6SS genes, a number of genes were found to be located in these regions (Figure 3). These included SciE (ImpE family protein), SciT protein, OmpA, a serine threonine kinase protein, a phosphoprotein phosphatase, TPR repeat protein, transposase, and insertion element IS-4. Out of these, SciE, SciT and OmpA were present only in the virulent species of the organisms.

ImpE family proteins are known to be involved in a temperature dependent secretion of various organisms. This gene was present within the genomic island regions of 17 organisms which included *V. fulvinicus* and the various species of Pseudomonas, Salmonella and Burkholderia. Based on the Pfam family information, this protein is known to interact with and manipulate eukaryotic cells, although the exact mechanism is not known.

OmpA, an outer membrane protein, was seen in the genomic islands of all species of *E. coli*, Yersinia, Pseudomonas, Burkholderia, Xanthomonas, Ralstonia, Hahella and Mesorhizobium. OmpA is known to be responsible for the translocation of effector molecules [12]. This gene is important for outer membrane stability and is composed of three functional domains including a hydrophilic extracellular mass, a beta-barrel transmembrane structure, and a peptidoglycan binding domain [12].

A Serine threonine kinase protein and a phosphoprotein phosphatase were seen to be present in the compositionally distinct regions in a few organisms which included species of Pseudomonas, Vibrio, Yersinia, Burkholderia, Xanthomonas, Hahella, Geobacter and Mesorhizobium. Literature survey shows that a serine threonine phosphorylation event is required for the initial activation of the T6SS in *P. aeruginosa,* which requires both a serine-threonine kinase and a protein phosphatase along with an FHA containing protein [3]. One of the components of T6SS, namely, VCA0112, had the FHA domain, indicating that the identified Serine Threonine kinase and phosphoprotein phosphatase might play a role in the initial activation even of the T6SS machinery.

## Discussion

### Identification of T6SS in Bacterial Genomes

All the organisms having orthologs of at least 10 of the known T6SS components were considered to have T6SS machinery. Our analysis indicated presence of T6SS in 42 bacterial species, all belonging to the proteobacteriaceae family. However, some of the pathogenic organisms belonging to the proteobacteriaceae family were not predicted to have T6SS. Examples of these included pathogens like Legionella, Klebsiella and Enterobacteria, which had orthologs of only 4, 8 and 9 T6SS components respectively. This implies that the pathogenicity of these organisms is dependent on some other factors. For example, the type III secretion system in Klebsiella, type IV secretion system in Legionella and types II and III secretion systems in Enterobacter, might be responsible for the pathogenicity of these organisms. The discordant distribution of T6SS genes across proteobacteriaceae family clearly indicates that there must have been gene(s) loss in certain organisms.

### Functional Classification of T6SS Components

**Figure 4:**
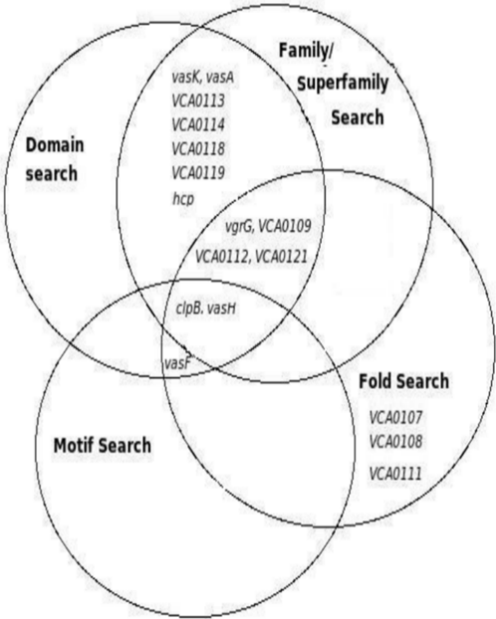
Functional annotation of Type VI Secretion System components based on domain, motifs, family and three dimensional fold searches. Genes at the intersections represent that they have been identified and annotated by two or more methods. For example VasF was identified by both fold and motif searches, whereas ClpB was identified using family, fold and motif searches.

Figure 4 illustrates genes which were annotated based on domain, motif, family and fold searches. While few of the T6SS components were identified by only one of the methods, others were picked by two or more searches.

Based on the function, T6SS components were characterized as structural, regulatory, effector and chaperon proteins. Except for VasH, which is known to be a Sigma 54 activating protein and a transcriptional regulator for the T6SS gene cluster [2], all the other components of T6SS could be grouped under three heads, based on their location in the cell, namely, those belonging to the outer membrane, transmembrane and inner membrane (Table 2). Some of these components comprised of the puncturing device.

**Table 2:** Putative functions assigned to various components of Type VI Secretion System.

Gene	Known function	Assigned function	Derived from the study
*vasA*	Structural protein	Inner membrane protein, involved in intramacrophagal replication	Orthology, domains, family and literature
*vasF*	Structural protein	Membrane-outer similar porin, transmembrane protein	Orthology, domains, family, superfamily, fold and literature.
*vasK*	Structural protein	IcmF-like structural protein, involved in cell-surface recognition resulting in adherence to epithelial cells.	Orthology, domains, family, superfamily and literature.
*VCA0107*	Unknown	Implicated in pathogenicity and secretion	Orthology, domains, family, fold and literature.
*VCA0109*	Unknown	Cytoplasmic protein IcmF related.	Orthology, domains, family, fold and literature.
*VCA0111*	Unknown	Cytoplasmic protein.	Orthology, family and fold search.
*VCA0112*	Unknown	FHA domain containing protein, important in activation of T6SS machinery.	Orthology, domains, family, superfamily and literature.
*VCA0114*	Unknown	Unknown	Orthology, domains, family and literature
*VCA0113*	Unknown	Lipoprotein outer membrane protein	Orthology, domains, family and literature
*VCA0118*	Unknown	Exported protein, involved in transmembrane activities.	Orthology, domains, family and literature
*VCA0121*	Unknown	Innermembrane protein	Orthology, domains, family, superfamily, fold and literature.
*VCA0122*	Unknown	Unknown	-
*VCA0119*	Unknown	Inner membrane protein, associated with exported proteins and similar to ImpA	Orthology, domains, family and literature
*vasH*	Regulatory protein-sigma 54	Transcriptional DNA binding, Sigma 54 protein	Orthology, domains, family, superfamily, fold and literature.
*hcp*	Effector protein	Hemolysin coregulated protein, responsible for cytotoxicity, an effector molecule also responsible for secretion of other effectors.	Orthology, Domains, family, superfamily, fold and literature.
*VgrG*	Effector protein	Forms a puncturing device-channel between outer membrane and host cell membrane	Orthology, domains, family, superfamily, fold and literature.
*ClpB*	Chaperone	ATP-binding protease, chaperone	Orthology, domains, family, superfamily, fold and literature.
*ompA*	Structural protein	Outer membrane protein, involved in translocation of effector molecules.	Phylogenetic clusters, protein-protein association, 3D fold and Genomic Island study
*ClpV*	Chaperone	Adaptor protein to clpB.	Phylogenetic cluster studies
*VC0893*	Chemotaxis protein	Chemotaxis protein interacting with vasF	Protein-protein association studies.
*sciE*	ImpE family protein	Associated with the T6SS components, implicated in protein secretion	Genomic Island study
Serine Threonine Kinase	Kinase	Interacts with FHA domain and involved in initial activation of T6SS machinery	Genomic Island study
Phospho protein phodphatase	Phosphatase	Interacts with FHA domain and involved in initial activation of T6SS machinery	Genomic Island study

### Puncturing Device

VgrG is known to be a Rhs element containing protein and is a cytotoxic effector molecule [9], which was also confirmed by its domain and family analyses. VgrG has three major domains, an Actin Cross linking domain (ACD), a peptidoglycan domain and a gp5 like domain. These domains in VgrG are known to be similar to the phage T4 spike protein, which is involved in puncturing of the host cell and formation of the core complex [9]. It has been suggested to be involved in formation of trimeric channel connecting the outer membrane and the host cell membrane and thus acting as a puncturing device of the T6SS machinery [9]. On the other hand, Hcp protein which is a hemolysin coregulated protein is an important effector protein of the T6SS. Hcp is a heme coregulated protein which forms a hexameric ring like structure that is thought to be involved in the formation of a transportation channel connecting the inner and outer membrane of the bacterium [2], [9]. Thus the two proteins, VgrG and Hcp, are suggested to form a continuous channel from inner membrane to outer membrane and outer membrane to the host membrane, with VgrG extending as a puncturing device into the host cell.

VCA0109, which was annotated as hypothetical protein, belonged to the lysozyme family protein that contained a gp25 domain which is known to hydrolyse NAG and NAM of peptidoglycan residues of the cell wall (host cell wall). A similar domain gp27 is known to participate in the assembly of the needle apparatus in T4 tail spike protein [9]. It is known that gp5 and gp27 domains of T4 tail spike protein are both required for the formation of the needle apparatus, gp5 being the base on which gp27 is placed [9]. The absence of gp27 domain in VgrG could thus be compensated by the gp25 domain of the VCA0109 protein. Thus gp5 domain of VgrG was proposed to interact with VCA0109 (which is similar to gp27 of the tail spike protein), VCA0109 functioning as a base plate assembly protein.

ClpB is known to have ATPase domains and clp chaperonin domain, and is known to hydrolyze ATP to provide energy [2], [9]. Although, ClpB does not belong to the puncturing machinery of T6SS, it also forms a hexameric ring like structure which interacts with Hcp and provides the energy required for the transport of the T6SS effector molecules [9]. Thus it could be hypothesized that in T6SS, ClpB interacts with Hcp, which in turn forms a translocation channel along with VgrG and VgrG together with VCA109 form a device to puncture the host membrane.

### Outer Membrane Protein

VCA0113, a hypothetical protein, had a lipoprotein domain and was involved with the exported proteins. It is involved in the intracellular trafficking, secretion and transport. This was identified to be the only outer membrane T6SS protein.

### Transmembrane Proteins

VasF and VasK, possessing transmembrane domains and having coiled-coil structures, are known to be similar to IcmH and IcmF proteins. It is known that the proteins IcmF and IcmH help in translocating a pore in the host cell and help in intracellular cytotoxicity [13]. Thus, both VasF and VasK were identified as periplasmic proteins and played a role in translocation. Also, based on superfamily information, VasK belonged to a G-proteins superfamily which is a P-loop containing nucleoside triphosphate hydrolase superfamily. This suggested that in addition to translocation function, VasK might be involved in effector recognition and signal transduction, acting as an accessory protein.

3D fold search of the hypothetical protein, VCA0118 indicated it to be a coiled-coiled structure. In addition, this protein had a signal peptide domain as well as a transmembrane domain and thus could be a transmembrane protein, although the exact functioning could not be predicted.

### Inner Membrane Proteins

VasA was predicted to be an inner membrane protein and thought to participate in intracellular activities.

Similarly, VCA0111, annotated as hypothetical protein, belonged to transposase family and had a cytoplasmic domain and thus predicted to be an inner membrane protein and thought to participate in inner membrane activities.

Domain information of the hypothetical protein VCA0112 showed presence of FHA domain. Proteins having FHA domains are known to interact with serine-threonine kinase, which in turn activates T6SS in Pseudomonas [3]. Thus, VCA0112 could play a role in initial activation, since a serine threonine protein is always seen to be associated with the T6SS proteins. This gene was identified as one of the additional genes in the compositionally distinct regions containing T6SS components.

Hypothetical proteins, VCA0119 and VCA0121 belonged to the ImpA family. Since ImpA related proteins are known to be inner membrane proteins which are associated with the exported proteins, VCA0119 and VCA0121 could be though as inner membrane protein associated with effector proteins of T6SS.

### Comparison of T6SS with Other Secretion Systems

From the literature as well as from the present study, it can be inferred that the T6SS machinery is similar to all the 3 types of sec-independent pathways, namely T1SS, T3SS and T4SS. For instance, all the four secretion systems (T1SS, T3SS T4SS and T6SS) transport proteins that lack an N-terminal signal sequence and use a sec independent transport for translocation of proteins. Also, all these systems extend from the inner membrane, through the periplasmic space and upto the outer membrane. Similarly, while YscN functions as the ATPase in T3SS, ClpB performs this job in T6SS, since both share similar domains and belong to a P-loop containing nucleoside triphosphate hydrolases superfamily. In the same way, while ClpB is the chaperone of the T6SS, InvB (CesD) acts as the T3SS chaperone. In terms of mechanism, T3SS is known to function by entering the host cell by puncturing through its needle formed by YscF. In a similar fashion, T6SS functions by puncturing the host cell with the help of its protein VgrG which is similar to T4 phage tail spike protein [9]. T6SS is also similar to T4SS since the transmembrane proteins VasF and VasK in T6SS share high sequence similarity to IcmF and IcmH of T4SS. On the other hand, the channel formed by HlyA and RtxA of T1SS is similar to that formed by VgrG and Hcp in T6SS. Thus, since T6SS components shared similarities to various components of T1SS, T3SS and T4SS, it was proposed that T6SS has adapted a mechanism, which is a fusion of all three systems.

### Proposed Model and Mechanism of T6SS

**Figure 5:**
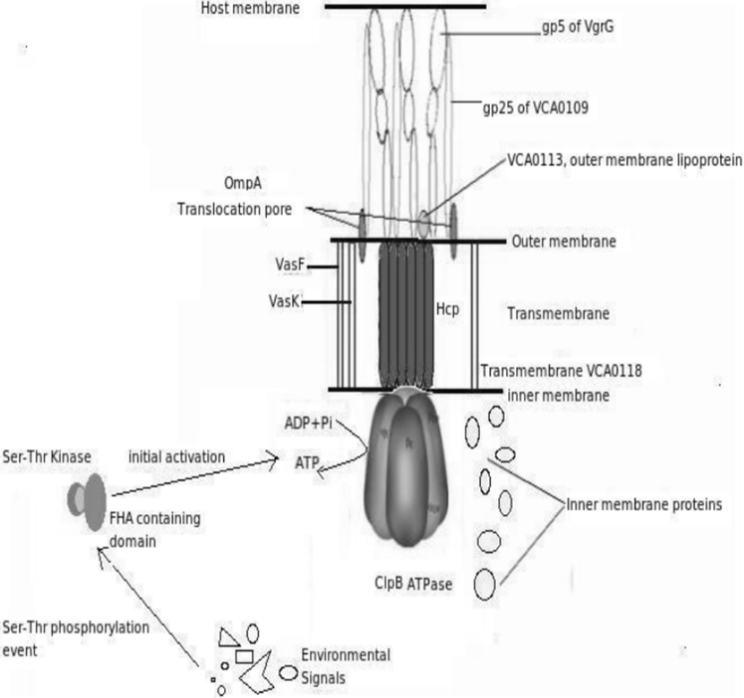
Proposed model of Type VI Secretion System. The figure represents the various components involved in its machinery and how they interact with each other.

Combining all the information from the different studies performed, following model of T6SS has been predicted and is schematically shown in Figure 5.

The initial activation of the system depends on environmental factors, which are not known. After appropriate signals are sensed by the system, VCA0112, having a FHA domain brings about the initial activation event on interaction with a serine threonine kinase. Once activated, the assembly of the T6SS machinery occurs, where the translocation channel is formed by Hcp, VCA0109 and VgrG. While Hcp forms a hexameric translocation channel in the transmembrane region, VgrG forms a trimeric needle like structure on the bacterial cell surface that reaches the host cell and acts as a puncturing device. VCA0109 forms a channel from the bacterial outer membrane to the host membrane and interacts with the gp5 domain of each monomer of VgrG. Assembly of all the inner membrane proteins, namely, VCA0107, VCA0108, VCA0111, VCA0114, VCA0119, VCA0121 and VCA0122, also occur through an unknown mechanism. After the assembly of all the components, ClpB, which acts as as a chaperone and ATPase, forms a hexameric structure which interacts with Hcp to hydrolyse ATP in order to translocate of effector molecules. Accessory proteins VasK and VasF (icmF family proteins) bring about cell surface reorganization, resulting in increased adherence to host cells. VasK also helps in recognition of the transport signal, thereby increasing the efficiency of translocation. The effector proteins (Hcp, VgrG, etc), with the help of the outer membrane associated lipoprotein VCA0113, pass through the translocation pore formed by the protein OmpA. All The inner membrane proteins mentioned above assist in stabilizing the complex and in bringing about other intracellular activities essential for the functioning of the T6SS. The transcriptional control of the T6SS machinery is probably brought about by VasH, a sigma-54 family protein. The system can be thought to be involved in a temperature dependent secretion, as it has proteins belonging to the imp family, which is known to be associated in a temperature dependent secretion.

Comparison of Figures 2 and 5 shows that most of the proteins having similar phylogenetic profiles (Figure 2) are seen to be physically interacting in the proposed model (Figure 5). These include five protein pairs, namely, (VasF, VasK), (VCA0113, VgrG), (VgrG, VCA0109), (Hcp, ClpB) and (VCA113, VCA0109), the phylogenetic profile difference between each of the protein-pairs being, 1 bit for the first two pairs, 2 bits for the third pair and 3 bits for the last two pairs, respectively. This observation is in line with the fact that co-evolving proteins, if interacting physically, may be under stronger selective pressure to conserve.

## Methods

**Figure 6:**
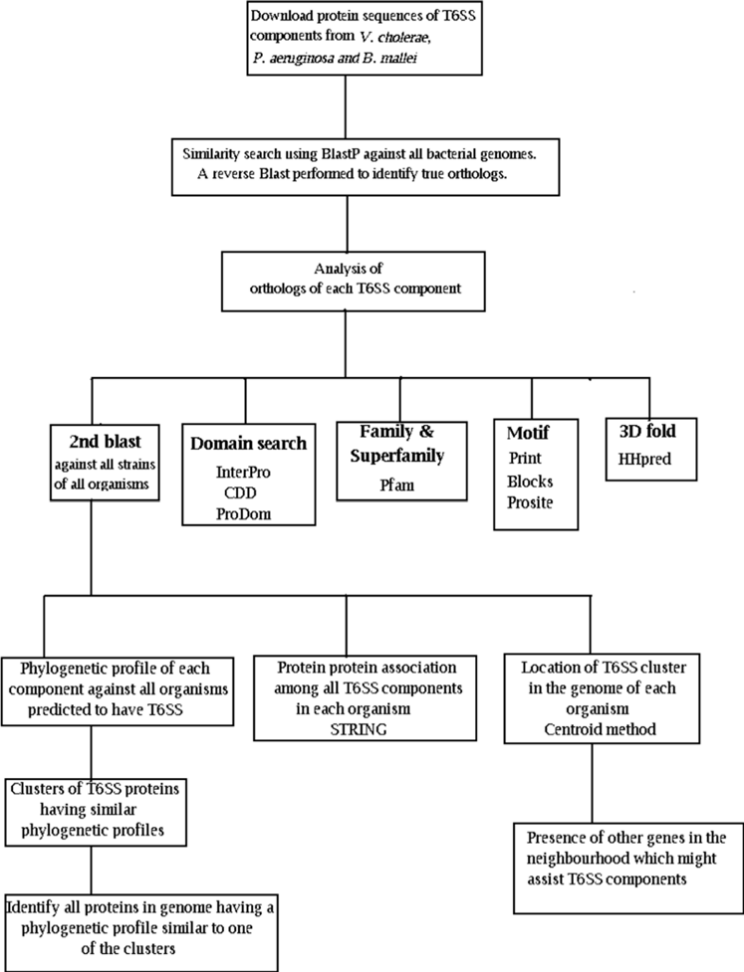
Flow chart depicting the various methodologies and analysis performed on known Type VI Secretion System components.

The protein sequences corresponding to all the genes which have been identified experimentally to be components of type VI secretion system in *V. cholerae, P. aeruginoasa* and *B. mallei* [2], [3], [4] were downloaded from NCBI (http://www.ncbi.nlm.nih.gov/). These comprise of the 18, 16 and 14 genes in the T6SS cluster of *V. cholerae, P. aeruginosa* and *B. mallei,* respectively. Figure 6 depicts the various analysis performed on each of the T6SS components.

### Orthology Search and Phylogenetic Profiling

Genes belonging to the previously known secretion systems (Type I through type V) have been seen to be conserved across all bacterial species possessing them [1]. In order to identify if this pattern is followed in T6SS, all the genomes in NCBI were searched against all the T6SS genes of the *V. cholerae, P. aeruginoasa and B. mallei* using the online NCBI BLAST search (http://www.ncbi.nlm.nih.gov/BLAST). Only those hits having homology with its corresponding T6SS gene in at least one of the above three organisms were considered and the orthologs were identified using reverse-BLAST. Table S1 lists bit scores and E-values of all the orthologs of the known T6SS components in all organisms. All organisms having orthologs of at least 10 known T6SS components were considered to possess T6SS and are listed in Table 1. In order to identify the complete set of T6SS components in all the species of each organism, each of the T6SS component identified in a particular species of an organism was then used to search for their orthologs using a second blast search against its other species for which complete genome sequences were known.

In order to identify genes which were widely represented across all organisms, a phylogenetic profile, which is matrix of the presence/absence of genes across all organisms, was created (Table S2). While a score of 0 was put for those cases where no orthologs were found, bit scores were considered wherever orthologs were identified. The orthologs for each of the components in the 3 organisms which had the best bit score were considered and the score of 1 was given to represent presence of the component.

Since the proteins which participate in the same pathway tend to show a similar pattern of inheritance and co-evolution and hence a similar phylogenetic profile [5], we analyzed the phylogenetic profile matrix (Table S2) of each of the T6SS protein. T6SS components were clustered based on the similarity of their profiles (Figure 2). While describing the phylogenetic profiles, the term ‘bit’ was used as defined by Pellegrini *et al,* 1999 [5]. Neighborhood-space consisted of all profiles which were different to each other by a maximum of 4 bits i.e their pattern of inheritance differed in a maximum of 4 organisms. It was important to consider the neighborhood-space, since apart from the proteins having the same profile, proteins belonging to the same neighborhood space were also considered to be functionally linked [5], [6]. Once the clusters were formed, analysis of all other proteins in each organism, belonging to one of these clusters was done. Such an analysis was likely to reveal those proteins which are associated with the T6SS machinery directly or indirectly, but were not picked up as orthologs in the homology search.

To probe the ancestry of the T6SS and to understand their evolutionary patterns, 16S rRNA sequences corresponding to all the completely sequenced proteobacteria were downloaded from RNA database Project II (http://wdcm.nig.ac.jp/RDP/html/index.html) and a 16S rRNA phylogenetic tree was constructed (Figure 1) using *MEGA* version 4 [14]. The tree was bootstrapped 500 times (i.e. generating 500 replicates) to ensure statistical confidence of the constructed tree.

### Protein Family and Domain Search

Since the GO annotation is not available for the Type VI secretion system, we searched for the protein family and domain annotations of the T6SS genes for their functional classification. While InterPro (http://www.ebi.ac.uk/InterProScan/), CDD (http://www.ncbi.nlm.nih.gov/Structure/cdd/wrpsb.cgi) and ProDOM (http://prodom.prabi.fr/prodom/current/html/form.php) databases were used for searching domains, Pfam database (http://pfam.sanger.ac.uk/) was used to search for protein families. We also looked for Superfamily (http://supfam.mrc-lmb.cam.ac.uk/SUPERFAMILY/) information for each protein.

### Motif Search

Presence of known motifs in the T6SS components was searched using the Motif Finder (http://gibk26.bse.kyutech.ac.jp/jouhou/HOMOLOGY/dbsearch/pdb/pdb_seq.html). Known patterns and motifs in these components were searched against the Prosite (http://www.expasy.ch/tools/scanprosite/), Block (http://blocks.fhcrc.org/) and Prints (http://www.bioinf.manchester.ac.uk/dbbrowser/PRINTS/) databases.

### Presence of Signal Peptides

In order to verify whether the proteins which are translocated by the T6SS possess any signal peptide, the presence of N/C-terminal peptides of the identified orthologs of effector molecules (Hcp & VgrG proteins in Vibrio) in all organisms were checked using the software SignalP [8].

### Detection of Protein Folds of T6SS Components

Proteins lacking significant sequence similarity sometimes have similar three dimensional structures, thereby suggesting similar functions. In order to identify the three dimensional folds of the T6SS components, a tool Hhpred (http://toolkit.tuebingen.mpg.de/hhpred) based on HMM-HMM comparison was used.

### Protein-Protein Association Network

To look for association partners among the components of the type VI secretion system, the web based tool called STRING (http://string.embl.de) which uses co-occurrence, gene neighborhood, gene fusion and text mining for predicting functionally related proteins, was used [15]. All the T6SS orthologs identified in each organism were searched to find interacting partners among them in order to identify components of the network that had been missed out by sequence similarity based methods.

### Location of T6SS in the Genomes

It has been observed that components of secretion systems Type I to Type IV are located in the regions of the genome which are compositionally distinct [16], [10]. In order to identify whether components of T6SS also belong to such regions, we analyzed the compositionally biased regions (Genomic Islands) in the genomes of the organisms using the Centroid method [11].

### Conclusion

We have predicted type VI secretion system in 42 proteobacteria based on our informatics study. The functions of the T6SS components were suggested based on the results of our analysis as well as information available from literature. The predicted mechanism underlying the secretion by T6SS needs to be experimentally validated.

## Supporting Information

Table S1 E-values and bit scores of the orthologs of the T6SS components. The protein names of the T6SS components correspond to that in Vibrio.
(0.24 MB DOC)

Table S2 Binary matrix showing the phylogenetic profile of each T6SS orthologs across all the organisms. The gene names are the corresponding names in V. cholerae T6SS components.
(0.15 MB DOC)

Table S3 T6SS orthologs in various strains for which its complete genome sequence is available.
(0.15 MB DOC)

Table S4 Conserved domains and families of the T6SS components.
(0.12 MB DOC)
