# Supplementary material for: Identification and Functional Characterization of Gene Components of Type VI Secretion System in Bacterial Genomes
Source: PLoS One. 2008 Aug 13;3(8):e2955. doi: 10.1371/journal.pone.0002955 (PMC2492809; doi:10.1371/journal.pone.0002955)
Supplement: Table S1 — E-values and bit scores of the orthologs of the T6SS components. The protein names of the T6SS components correspond to that in Vibrio. (0.24 MB DOC) [file pone.0002955.s001.doc]

**Table S1:** E-values and bit scores of the orthologs of the T6SS components. The protein names of the T6SS components correspond to that in Vibrio.

|  |  | **E-value** | **Bit score** |
| --- | --- | --- | --- |
| **Vibrio** | **VasA** | **0** | **1154** |
| Marinobacter | Hyp | 0 | 716 |
| Aeromonas | ASA_2467 | 9.00E-176 | 618 |
| Erwinia | ECA3442 | 7.00E-169 | 595 |
| Yersinia | YPTB3635 | 4.00E-164 | 580 |
| Pseudomonas | PP_4077 | 2.00E-152 | 540 |
| Photorhabdus | plu0369 | 3.00E-149 | 530 |
| Shigella | SSON_0250 | 9.00E-134 | 479 |
| Escherichia coli | Z0260 | 9.00E-134 | 479 |
| Geobacter | GSU0431 | 2.00E-099 | 364 |
| Burkholderia | BMAA0740 | 2.00E-078 | 295 |
| Ralstonia | Hyp | 1.00E-072 | 275 |
| Hahella | HCH_04251 | 2.00E-065 | 252 |
| Xanthomonas | XCV4238 | 1.00E-061 | 239 |
| Salmonella | Hyp | 2.00E-050 | 202 |
| Mesorhizobium | mlr2342 | 4.00E-042 | 168 |
| Shewanella | Hyp | 5.00E-053 | 211 |
| Photobacterium | PBPRA0671 | 9.00E-052 | 200 |
|  |  |  |  |
| **Vibrio** | **VasK** | **0** | **2311** |
| Marinobacter | Maqu_3720 | 0 | 1004 |
| Aeromonas | AHA_1845 | 0 | 885 |
| Erwinia | ECA3432 | 0 | 825 |
| Pseudomonas | PP_4071 | 0 | 811 |
| Yersinia | YPTB3625 | 0 | 803 |
| Photorhabdus | plu0359 | 0 | 788 |
| Escherichia coli | Z0250 | 0 | 760 |
| Hahella | HCH_04298 | 8.00E-111 | 404 |
| Photobacterium | PBPRA0662 | 5.00E-106 | 388 |
| Mesorhizobium loti | mlr2360 | 2.00E-100 | 369 |
| Shewanella | Sfri_2373 | 3.00E-098 | 362 |
| Burkholderia | BMAA0399 | 3.00E-098 | 362 |
| Xanthomonas | XAC4119 | 6.00E-082 | 308 |
| Ralstonia | H16_A0663 | 2.00E-079 | 299 |
| Shigella | SSON_0236 | 2.00E-074 | 283 |
| Salmonella | STM0285 | 8.00E-074 | 281 |
|  |  |  |  |
| **Vibrio** | **VasF** | **2.00E-141** | **502** |
| Aeromonas | ASA_2462 | 4.00E-062 | 239 |
| Erwinia | ECA3437 | 2.00E-061 | 236 |
| Marinobacter | Maqu_3725 | 2.00E-060 | 234 |
| Pseudomonas | PP_2616 | 1.00E-048 | 194 |
| Yersinia | YPTB3630 | 1.00E-047 | 191 |
| Photorhabdus | plu0364 | 1.00E-045 | 184 |
| Shigella | SSON_0244 | 3.00E-045 | 183 |
| Escherichia coli | Z0255 | 4.00E-045 | 182 |
| Burkholderia | BMAA0398 | 5.00E-029 | 129 |
| Hahella | HCH_04299 | 2.00E-027 | 124 |
| Shewanella | Sfri_2374 | 1.00E-026 | 121 |
| Xanthomonas | XCV2137 | 7.00E-026 | 119 |
| Photobacterium | PBPRA0661 | 2.00E-025 | 117 |
| Ralstonia | H16_A0662 | 2.00E-018 | 94.4 |
| Salmonella | STM0282 | 5.00E-017 | 89.7 |
| Mesorhizobium | mlr2348 | 7.00E-011 | 69.3 |
| Geobacter |  |  |  |
|  |  |  |  |
| **Vibrio** | **VasH** | **0** | **961** |
| Marinobacter | Maqu_3723 | 5.00E-101 | 370 |
| Aeromonas | ASA_2460 | 2.00E-089 | 332 |
| Erwinia | ECA3435 | 3.00E-087 | 324 |
| Shewanella | Sfri_1976 | 2.00E-063 | 244 |
| Geobacter | Gmet_3263 | 2.00E-061 | 238 |
| Ralstonia | Reut_A1698 | 9.00E-061 | 236 |
| Shigella | SSON_3020 | 2.00E-060 | 235 |
| Escherichia coli | Z4208 | 2.00E-060 | 235 |
| Photorhabdus | plu3311 | 3.00E-060 | 234 |
| Xanthomonas | XAC0226 | 1.00E-059 | 233 |
| Salmonella | STM4174 | 8.00E-059 | 229 |
| Pseudomonas | PSPTO_5424 | 3.00E-063 | 244 |
| Mesorhizobium | mll3001 | 2.00E-055 | 215 |
| Burkholderia | BMA0037 | 1.00E-057 | 222 |
| Photobacterium | PBPRA0918 | 2.00E-057 | 222 |
| Hahella | HCH_05199 | 2.00E-058 | 225 |
| Yersinia | YPTB2874 | 8.00E-058 | 223 |
|  |  |  |  |
| **Vibrio** | **VgrG** | **0** | **2000** |
| Aeromonas | Hyp | 0 | 666 |
| Erwinia | ECA3427 | 6.00E-178 | 626 |
| Marinobacter | Hyp | 5.00E-173 | 610 |
| Photorhabdus | plu0355 | 3.00E-132 | 474 |
| Yersinia | YPTB3284 | 1.00E-128 | 462 |
| Pseudomonas | Hyp | 5.00E-122 | 441 |
| Escherichia coli | Hyp | 3.00E-110 | 402 |
| Shigella | Hyp | 3.00E-110 | 401 |
| Mesorhizobium | mll2357 | 4.00E-072 | 272 |
| Burkholderia | BMAA0410 | 1.00E-081 | 304 |
| Photobacterium | PBPRA0675 | 6.00E-064 | 245 |
| Hahella | HCH_02182 | 4.00E-083 | 309 |
| Xanthomonas | XAC4124 | 5.00E-059 | 229 |
| Ralstonia | Reut_A1726 | 5.00E-078 | 292 |
| Geobacter | Gmet_0286 | 3.00E-085 | 316 |
| Shewanella | Sfri_2360 | 3.00E-067 | 256 |
| Salmonella | STM0289 | 2.00E-065 | 250 |
|  |  |  |  |
| **Vibrio** | **VCA0107** | **2.00E-087** | **322** |
| Marinobacter | Maqu_3733 | 5.00E-057 | 221 |
| Aeromonas | ASA_2470 | 6.00E-053 | 207 |
| Erwinia | ECA3445 | 5.00E-052 | 204 |
| Geobacter | Gmet_0278 | 7.00E-042 | 171 |
| Ralstonia | Reut_B5266 | 1.00E-025 | 116 |
| Shigella | SSON_0254 | 1.00E-044 | 179 |
| Escherichia coli | Z0264 | 1.00E-042 | 173 |
| Photorhabdus | plu0372 | 3.00E-051 | 201 |
| Xanthomonas | Hyp | 2.00E-017 | 89.4 |
| Salmonella | STM0273 | 2.00E-008 | 54.7 |
| Pseudomonas | Pput_2127 | 3.00E-043 | 175 |
| Mesorhizobium | mlr2337 | 5.00E-013 | 75.1 |
| Burkholderia | BMAA0744 | 2.00E-041 | 169 |
| Photobacterium | PBPRA0666 | 2.00E-015 | 83.2 |
| Hahella | HCH_04247 | 8.00E-013 | 74.3 |
| Yersinia | YPTB3638 | 7.00E-050 | 197 |
|  |  |  |  |
| **Vibrio** | **VCA0108** | **0** | **988** |
| Marinobacter | Hyp | 0 | 819 |
| Aeromonas | AHA_1833 | 0 | 789 |
| Erwinia | ECA3444 | 0 | 790 |
| Geobacter | Hyp | 0 | 667 |
| Ralstonia | Hyp | 4.00E-124 | 446 |
| Shigella | Hyp | 1.00E-106 | 389 |
| Escherichia coli | Z0262 | 0 | 692 |
| Photorhabdus | plu0371 | 0 | 773 |
| Xanthomonas | XAC4146 | 2.00E-095 | 351 |
| Salmonella | Hyp | 2.00E-095 | 351 |
| Pseudomonas | Hyp | 0 | 710 |
| Mesorhizobium | mlr2338 | 3.00E-092 | 335 |
| Burkholderia | BMAA0743 | 2.00E-179 | 630 |
| Photobacterium | PBPRA0667 | 2.00E-089 | 325 |
| Hahella | HCH_04248 | 1.00E-083 | 306 |
| Yersinia | YPTB3637 | 0 | 780 |
|  |  |  |  |
| **Vibrio** | **VCA0109** | **6.00E-077** | **286** |
| Marinobacter | Hyp | 2.00E-023 | 108 |
| Aeromonas | ASA_2468 | 1.00E-019 | 96.3 |
| Erwinia | ECA3443 | 5.00E-020 | 97.8 |
| Yersinia | YPTB3636 | 2.00E-018 | 92.4 |
| Pseudomonas | PP_26222 | 3.00E-012 | 72.0 |
| Photorhabdus | plu0370 | 4.00E-022 | 104 |
| Shigella | Hyp | 2.00E-008 | 55.5 |
| Escherichia coli | Z0261 | 2.00E-014 | 79.0 |
| Geobacter | Hyp | 3.00E-008 | 58.5 |
| Ralstonia | Reut_B5259 | 0.008 | 37.4 |
| Hahella | HCH_04250 | 0.02 | 39.7 |
| Xanthomonas | Hyp | 0.029 | 35.4 |
| Salmonella | Hyp | 5.00E-017 | 89.7 |
| Mesorhizobium | mlr6561 | 0.72 | 30.8 |
| Photobacterium | Hyp | 2.00E-015 | 83.2 |
|  |  |  |  |
| **Vibrio** | **VCA0111** | **0** | **651** |
| Marinobacter | Maqu_3729 | 5.00E-077 | 289 |
| Aeromonas | ASA_2466 | 2.00E-058 | 227 |
| Erwinia | ECA3441 | 3.00E-069 | 263 |
| Geobacter | Gmet_3314 | 1.00E-021 | 105 |
| Ralstonia | Reut_B5274 | 4.00E-013 | 77.4 |
| Shigella | SSON_0249 | 3.00E-054 | 213 |
| Escherichia coli | Z0259 | 1.00E-054 | 214 |
| Photorhabdus | plu0368 | 7.00E-055 | 216 |
| Xanthomonas | XAC4141 | 5.00E-021 | 103 |
| Salmonella | STM0267 | 2.00E-012 | 75.1 |
| Pseudomonas | PP_4078 | 1.00E-057 | 224 |
| Mesorhizobium | mlr2343 | 2.00E-008 | 62.0 |
| Burkholderia | BMAA0739 | 1.00E-017 | 92.4 |
| Photobacterium | PBPRA0672 | 3.00E-007 | 51.2 |
| Hahella | HCH_04252 | 2.00E-005 | 45.1 |
| Yersinia | YPTB3634 | 2.00E-056 | 221 |
|  |  |  |  |
| **Vibrio** | **VCA0112** | 0 | 929 |
| Marinobacter | Maqu_3728 | 5.00E-061 | 237 |
| Aeromonas | ASA_2465 | 1.00E-042 | 176 |
| Erwinia | ECA3440 | 1.00E-040 | 169 |
| Yersinia | YPTB3633 | 2.00E-041 | 172 |
| Pseudomonas | PP_2619 | 1.00E-013 | 80.1 |
| Photorhabdus | plu0367 | 8.00E-050 | 199 |
| Shigella | SSON_0248 | 1.00E-042 | 176 |
| Escherichia coli | Z0258 | 4.00E-043 | 177 |
| Burkholderia | BMAA0393 | 0.041 | 36.6 |
| Hahella | HCH_04302 | 1.00E-008 | 63.2 |
| Xanthomonas | XOO_2894 | 1.00E-005 | 53.1 |
| Mesorhizobium | mlr2345 | 5.00E-008 | 56.2 |
| Shewanella | Sfri_2377 | 4.00E-012 | 74.7 |
| Photobacterium | PBPRA0658 | 6.00E-005 | 50.8 |
|  |  |  |  |
| **Vibrio** | **VCA0113** | 3.00E-087 | 321 |
| Marinobacter | Maqu_3727 | 7.00E-027 | 120 |
| Aeromonas | ASA_2464 | 6.00E-028 | 124 |
| Erwinia | ECA3439 | 1.00E-027 | 123 |
| Yersinia | YPTB3632 | 1.00E-022 | 107 |
| Pseudomonas | PP_4079 | 1.00E-014 | 80.5 |
| Photorhabdus | plu0366 | 1.00E-024 | 113 |
| Shigella | SSON_0247 | 4.00E-017 | 88.6 |
| Escherichia coli | Z0257 | 3.00E-017 | 89.0 |
| Burkholderia | BMAA1912 | 2.00E-005 | 49.7 |
| Ralstonia | H16_A0660 | 7.00E-010 | 64.3 |
| Hahella | HCH_04301 | 7.00E-010 | 64.3 |
| Xanthomonas | XOO3483 | 1.3 | 28.9 |
| Salmonella | STM0280 | 3.00E-013 | 75.5 |
| Mesorhizobium | mlr2346 | 4.00E-005 | 43.9 |
| Shewanella | Sfri_2376 | 2.00E-005 | 49.3 |
| Photobacterium | PBPRA0659 | 1.00E-008 | 60.5 |
|  |  |  |  |
| **Vibrio** | **VCA0114** | **0** | **881** |
| Marinobacter | Maqu_3726 | 4.00E-167 | 589 |
| Aeromonas | ASA_2463 | 2.00E-155 | 550 |
| Erwinia | ECA3438 | 4.00E-154 | 546 |
| Yersinia | YPTB3631 | 1.00E-119 | 431 |
| Pseudomonas | PP_2617 | 3.00E-126 | 453 |
| Photorhabdus | plu0365 | 1.00E-107 | 391 |
| Shigella | SSON_0245 | 4.00E-063 | 243 |
| Escherichia coli | Z0256 | 1.00E-103 | 378 |
| Burkholderia | BMAA0397 | 1.00E-088 | 328 |
| Ralstonia | H16_A0661 | 2.00E-077 | 291 |
| Hahella | HCH_04300 | 3.00E-103 | 377 |
| Xanthomonas | XAC4121 | 2.00E-104 | 381 |
| Salmonella | STM0281 | 3.00E-070 | 267 |
| Mesorhizobium | mlr2347 | 6.00E-050 | 200 |
| Shewanella | Sfri_2375 | 7.00E-085 | 316 |
| Photobacterium | PBPRA0660 | 1.00E-082 | 308 |
|  |  |  |  |
| **Vibrio** | **VCA0118** | **5.00E-118** | **424** |
| Yersinia | YPTB3627 | 1.00E-009 | 64.7 |
| Pseudomonas | PP_4073 | 1.00E-013 | 78.2 |
| Photorhabdus | plu0361 | 3.00E-012 | 73.6 |
| Escherichia coli | Z0253 | 1.00E-011 | 71.6 |
|  |  |  |  |
|  |  |  |  |
| **Vibrio** | **VCA0119** | **0** | **849** |
| Marinobacter | Maqu_3721 | 3.00E-077 | 291 |
| Aeromonas | AHA_1844 | 7.00E-059 | 229 |
| Erwinia | ECA3433 | 2.00E-058 | 228 |
| Yersinia | YPTB3626 | 5.00E-062 | 240 |
| Pseudomonas | PP_2626 | 1.00E-037 | 159 |
| Photorhabdus | plu0360 | 2.00E-050 | 202 |
| Shigella | SSON_0240 | 8.00E-052 | 206 |
| Escherichia coli | Z0251 | 3.00E-037 | 157 |
| Geobacter | Gmet_0275 | 2.00E-009 | 65.5 |
| Burkholderia | BMAA0747 | 2.00E-005 | 48.5 |
| Ralstonia | Reut_B5272 | 7.00E-011 | 70.5 |
| Photobacterium | PBPRA0665 | 0.16 | 35.8 |
|  |  |  |  |
| **Vibrio** | **VCA0121** | **0** | **771** |
| Erwinia | ECA3431 | 2.00E-007 | 58.5 |
| Yersinia | YPTB3624 | 3.00E-012 | 74.7 |
| Photorhabdus | plu0335 | 5.00E-010 | 67.4 |
| Shigella | SSON_0234 | 2.00E-010 | 68.6 |
| Escherichia coli | Z0249 | 2.00E-010 | 68.6 |
| Hahella | HCH_03070 | 5.8 | 27.7 |
| Mesorhizobium | mll7563 | 0.69 | 30.8 |
|  |  |  |  |
| **Vibrio** | **ClpB** | **0** | **1594** |
| Marinobacter | Maqu_0880 | 0 | 1184 |
| Aeromonas | ASA_0246 | 0 | 1282 |
| Erwinia | ECA3345 | 0 | 1231 |
| Yersinia | YPTB0848 | 0 | 1242 |
| Pseudomonas | Pput_0665 | 0 | 1139 |
| Photorhabdus | plu1270 | 0 | 1237 |
| Shigella | SSON_2718 | 0 | 1244 |
| Escherichia coli | Z3886 | 0 | 1244 |
| Geobacter | Gmet_2851 | 0 | 897 |
| Burkholderia | Hyp | 0 | 1079 |
| Ralstonia | H16_A2249 | 0 | 1047 |
| Hahella | HCH_05918 | 0 | 1185 |
| Xanthomonas | XCV3324 | 0 | 1129 |
| Salmonella | STM2660 | 0 | 1254 |
| Mesorhizobium | mll3429 | 0 | 908 |
| Shewanella | Sfri_0669 | 0 | 1261 |
| Photobacterium | PBPRA3018 | 0 | 1367 |
|  |  |  |  |
| **Vibrio** | **Hcp** | **1.00E-098** | **358** |
| Marinobacter | Maqu_0180 | 2.00E-078 | 290 |
| Aeromonas | AHA_1118 | 5.00E-078 | 290 |
| Erwinia | ECA2866 | 4.00E-077 | 287 |
| Yersinia | YPTB3639 | 1.00E-076 | 285 |
| Pseudomonas | Pput_2118 | 2.00E-061 | 235 |
| Photorhabdus | plu0373 | 7.00E-077 | 286 |
| Shigella | SSON_0255 | 1.00E-071 | 269 |
| Escherichia coli | Z0266 | 2.00E-072 | 271 |
| Geobacter | Gmet_0280 | 1.00E-022 | 106 |
| Burkholderia | BMAA0742 | 6.00E-004 | 42.4 |
| Ralstonia | Reut_B5261 | 3.00E-025 | 115 |
| Xanthomonas | XOO3479 | 0.65 | 30.8 |
| Salmonella | STM3131 | 0.014 | 37.7 |
| Mesorhizobium | mlr2339 | 1.00E-005 | 48.1 |
