# Supplementary material for: Identification and Functional Characterization of Gene Components of Type VI Secretion System in Bacterial Genomes
Source: PLoS One. 2008 Aug 13;3(8):e2955. doi: 10.1371/journal.pone.0002955 (PMC2492809; doi:10.1371/journal.pone.0002955)
Supplement: Table S3 — T6SS orthologs in various strains for which its complete genome sequence is available. (0.15 MB DOC) [file pone.0002955.s003.doc]

**Table S3 :**  T6SS orthologs in various strains for which its complete genome sequence is available.

|  | **Virulent strains** | **No: of T6SS homologs** | **Avirulent strains** | **No: of T6SS homologs** |
| --- | --- | --- | --- | --- |
| Vibrio | *V.cholerae* | 18 | *V.fischeri* | 0 |
|  | *V.parahemolyticus* | 18 | *V.harvey* | 0 |
|  | *V.fulvinicus* | 18 |  |  |
|  |  |  |  |  |
| Erwinia | *E.caratovora* | 17 | - | - |
|  |  |  |  |  |
| Yersinia | *Y.pseudotuberculosis* | 17 |  |  |
|  | *Y.pestis* | 17 |  |  |
|  | *Y.enterocolitica* | 17* |  |  |
|  |  |  |  |  |
| E.coli | *E.coli* O157 | 18 | *E.coli* K12 | 0 |
|  | *E.coli* B171 | 18 | *E.coli* W3110 | 0 |
|  | *E.coli* CFT073 | 15 |  |  |
|  | *E.coli* 536 | 18 |  |  |
|  | *E.coli* APEC | 18 |  |  |
|  | *E.coli* UTI89 | 18 |  |  |
|  |  |  |  |  |
| Aeromonas | *A.hydrophila* | 15 |  |  |
|  | *A.salmonicida* | 15 |  |  |
|  |  |  |  |  |
| Shigella | *S.sonnei* | 18 |  |  |
|  | *S.flexneri^1^* | 13 |  |  |
|  | *S.dysentriae^1^* | 13 |  |  |
|  |  |  |  |  |
|  |  |  |  |  |
| Pseudomonas | *P.aeruginosa* | 16 | *P.stutzeri* | 0 |
|  | *P.syringae* | 16 | *P.putida* | 16 |
|  | *P.entomophila* | 16 | *P.fluorescens* | 16 |
|  |  |  |  |  |
| Photorhabdus | *P.luminescens* | 16 | - | - |
|  |  |  |  |  |
| Marinobacter |  |  | *M.aquaeolei* | 15 |
|  |  |  |  |  |
| Mesorhizobium |  |  | *M.loti* | 15 |
|  |  |  |  |  |
| Photobacterium | *P.profundum* | 15 | - | - |
|  |  |  |  |  |
| Xanthomonas | *X.campestris* | 14 |  |  |
|  | *X.oryzae* | 14 | *X.codiaei* | 0 |
|  | *X.axonpodis* | 14 |  |  |
|  |  |  |  |  |
| Ralstonia | *R.solanicearum* | 14 | *R.eutropa* | 14 |
|  | *R.metallidurans* | 14 |  |  |
|  |  |  |  |  |
| Hahella | *H.chejuensis* | 14 | - | - |
|  |  |  |  |  |
| Burkholderia | *B.malliei* | 14 | *B. thailandensis* | 0 |
|  | *B.pseudomallei* | 14 |  |  |
|  | *B.cepia* | 14 |  |  |
|  | *B.cenocepia* | 14 |  |  |
|  |  |  |  |  |
| Geobacter |  |  | *G.mettaliduricans* | 11 |
|  |  |  | *G.sulfurreducens* | 11 |
|  |  |  |  |  |
| Salmonella | *S.enterica* | 12 |  |  |
|  |  |  |  |  |
| Shewanella | *S.frigidimarina* | 10 |  |  |

^1^ strains picked up in the second Blast.
