# Supplementary material for: Identification and Functional Characterization of Gene Components of Type VI Secretion System in Bacterial Genomes
Source: PLoS One. 2008 Aug 13;3(8):e2955. doi: 10.1371/journal.pone.0002955 (PMC2492809; doi:10.1371/journal.pone.0002955)
Supplement: Table S4 — Conserved domains and families of the T6SS components. (0.12 MB DOC) [file pone.0002955.s004.doc]

**Table S4:** Conserved domains and families of the T6SS components

|  | **Domain1** | **Domain2** | **Domain3** | **Domain4** | **Domain5** | **Protein Family** |
| --- | --- | --- | --- | --- | --- | --- |
|  |  |  |  |  |  |  |
| **ClpB** | PR00300 clp chaperonein | SM00382 ATPase core | PF00004 ATPase core | PF07724 | PTHR11638 ATP dep | ATPase family associated with various cellular activities (AAA) |
| **VasA** | PF05947 DUF879 | PIRSF028304 UCP |  |  | Protease. | IcmF related prot |
| **VasF** | Transmembrane regions tmhmm |  |  |  |  | Porin transmembrane family |
| **VasH** | PF00158 sigma 54 activator | Ps00675 sigma54 interact_1 | PS00676 sigma54 interact_2 | PS00688 int_ 3 | PS500045 int_4 | Transcriptional DNA binding sigma 54 family |
| **VasK** | PF06761 ICMF related | DUF 06744 | SignalP signal peptide | Tmhmm transmembrane regions |  | Bacterial extracellular solute-binding protein, family 3, ImcF-related family |
| **VCA0107** | PIRSF028301 UCP | PF05591 DUF770 |  |  |  | Spectrin repeat family |
| **VCA0108** | PF05943 DUF8771 |  |  |  |  | Accessory gene regulator family, quorum sensing regulatory protein family (agrB) |
| **VCA0109** | PF04955 GPW_gp25 lysozyme | PIRSF028303 UCP |  |  |  | Gene 25-like lysozyme gpw_gp25 |
| **VCA0111** | PF06996 DUF1305 |  |  |  |  | Transposase, DNA topoisomerase type IA family |
| **VCA0112** | SSF49879 SMAD_FHA |  |  |  |  | The FHA (Forkhead-associated) family |
| **VCA0113** | PS51257 prokar_lipoprtn |  |  |  |  | VirB/Tra/Trw family, prok liporotein family |
| **VCA0114** | PF05936 DUF876 |  |  |  |  | FHC containing family, outer membrane |
| **VCA0118** | SignalP signal peptide | Tmhmm transmembrane region |  |  |  | Signal peptide and transmembrane family of proteins |
| **VCA0119** | PF06812 impA rel_N |  |  |  |  | ImpA related N terminal family, inner membrane |
| **VCA0121** | PF 06812 impA rel_N |  |  |  |  | ImpA related N terminal family, inner membrane |
| **VCA0122** | NO CD determined |  |  |  |  | Not detected |
| **VgrG** | PF04524 DUF 586 | TIGR01646 vgr_GE/ACD | PG_binding_1 | GPW_gp5 lysozyme |  | Rhs element Vgr protein family |
| **Hcp** | Pf05638 Hemolysin-coregulated protein (uncharacterized) [Function unknown]. |  |  |  |  | IPR008514 Virulence factor for secretion apparatus |
